# The Association between Circulating microRNAs and the Risk of Active Disease Development from Latent Tuberculosis Infection: a Nested Case-Control Study

**DOI:** 10.1128/spectrum.02625-21

**Published:** 2022-04-18

**Authors:** Henan Xin, Xuefang Cao, Ying Du, Jiaoxia Yan, Rui He, Zisen Liu, Haoran Zhang, Yijun He, Bin Zhang, Dakuan Wang, Ling Guan, Fei Shen, Boxuan Feng, Zhusheng Quan, Yongpeng He, Jianmin Liu, Qi Jin, Shouguo Pan, Lei Gao

**Affiliations:** a NHC Key Laboratory of Systems Biology of Pathogens, Institute of Pathogen Biology, and Center for Tuberculosis Research, Chinese Academy of Medical Sciences and Peking Union Medical College, Beijing, People’s Republic of China; b Center for Diseases Control and Prevention of Zhongmu, Zhengzhou, People’s Republic of China; c Sixth People’s Hospital of Zhengzhou, Zhengzhou, People’s Republic of China; Shandong First Medical University

**Keywords:** tuberculosis, latent tuberculosis infection, microRNA, biomarker, nested case-control study

## Abstract

Tuberculosis (TB) remains one of the deadliest communicable diseases. Biomarkers predicting the risk of active disease development from latent tuberculosis infection (LTBI) are urgently needed for precise intervention. This study aimed to identify potential circulating microRNAs (miRNAs) playing such a role in Chinese population. Based on a prospective study aiming to track the development of active TB among rural residents with LTBI, the baseline levels of circulating miRNAs were retrospectively compared between those who developed TB (case group) and those age-gender matched controls remain free of TB (contraol group) during the follow-up. Agilent human miRNA microarray were used to select differently expressed circulating miRNAs and verified by subsequent real-time quantitative PCR (RT-qPCR). Six candidate miRNAs were expressed at statistically significant levels between the two groups at the baseline, as determined by microarray. Following verification among 150 study participants by RT-qPCR, the levels of *hsa-miR-16-5p* (*P* < 0.001) and *hsa-miR-451a* (*P* < 0.001) were found to be significantly lower in case group compared to control group. The combined areas under curves (AUCs) and precision-recall curves (PRCs) were 0.84, 0.86 and 0.85, 0.87 for *hsa-miR-16-5p* and *hsa-miR-451a*, respectively. *hsa-miR-451a* combined with body mass index (BMI) and prior history of TB presented the best performance, with a sensitivity of 80.82% and an acceptable specificity of 79.22%. After adjusting the two co-variables, the AUC of *hsa-miR-451a* was 0.78. Circulating levels of *hsa-miR-451a* showed potential to predict development of active TB from LTBI in a Chinese population. Further studies are warranted to verify these findings in varied study settings.

**IMPORTANCE** Approximately a quarter of the world population are infected with M. tuberculosis and about 5% to 10% of these might develop active disease in their lifetime. Preventive treatment could effectively protect individuals at a high risk of developing active disease from LTBI, and is regarded as a critical component of End TB Strategies. Biomarkers which could accurately identify high-risk population and predict the risk of disease development are urgently needed for developing local guidelines of LTBI management and precise intervention. A nested case-control study was designed to explore possible microRNAs related with TB occurrence based on a previous prospective study, which aimed to track the development of active TB among rural residents with LTBI. The baseline circulating levels of *hsa-miR-16-5p* and *hsa-miR-451a* were significantly lower in TB cases compared to those in LTBI controls. Further receiver operator characteristic (ROC) curve analysis found that *hsa-miR-451a* showed considerable potential to predict the development of active TB from LTBI.

## INTRODUCTION

Although COVID-19 has overtaken tuberculosis (TB) as the deadliest communicable disease worldwide during the past year, TB remains a major global public health challenge. In 2019, it was estimated that 10 million people developed TB, with 1.4 million deaths. Approximately one-fourth of the world population is infected with M. tuberculosis *(M.tb)* and about 5 to 10% of infected persons might develop active disease during their lifetime (1). Therefore, individuals with latent TB infection (LTBI) are regarded as an enormous reservoir of TB. Preventive treatment has proven to be an effective tool for protecting individuals at high risk of developing active disease from LTBI and is now a critical component of End TB Strategies. Identifying individuals who might benefit from preventive therapy is crucial for precise intervention and is also a huge challenge. Currently, the recommended target populations for LTBI treatment are determined by evidence-based risk factors of developing active disease, such as with HIV infection, with close contact to infectious TB patients or using immunosuppressant (2). However, the distribution of the risk factors might vary in different populations. Exploring potential biomarkers which could accurately predict the risk of disease development is needed to develop local guidelines for LTBI management and precise intervention.

MicroRNAs (miRNAs) are well known to play important roles in regulating gene expression by targeting the mRNAs of protein-coding genes (3). Over the past decade, specific miRNAs have been identified which modulate innate immune responses, cytokine responses, and immune development (4, 5). Although the established functions of miRNAs are intracellular, numerous studies have detected highly stable extracellular circulating miRNAs, which can be easily accessed from blood (serum/plasma) (6). The role of circulating miRNAs as possible diagnostic biomarkers for TB has been widely studied. Sinigaglia et. al. (7) summarized the latest studies and reported miRNA signatures which could discriminate active TB patients from those with LTBI or with no *M.tb* infection. However, most published studies, including our previous study (8), have been based on case-control or cross-sectional study designs with limited sample sizes, and prospective studies have been rarely conducted. In our population-based, multicenter, prospective cohort study conducted in rural China, we followed individuals with baseline positive results for interferon gamma release assays (IGRA) or tuberculin skin tests (TST) (≥10 mm) for 5 years to track the development of active pulmonary TB (9). Based on this study, we intended to explore potential miRNA biomarkers which predict TB development from LTBI by retrospectively detecting and comparing baseline levels of circulating miRNAs between those who developed TB and those who remained free of TB (LTBI controls) using a nested case-control design.

## RESULTS

### Characteristics of study participants.

First, 20 TB cases and 20 age- and gender-matched LTBI controls with baseline IGRA+ and TST+ results were randomly selected for Agilent Human miRNA detection to estimate the baseline circulating levels of 2,549 miRNAs. Table 1 presents major baseline characteristics of these 40 participants, and no significant differences were found between the two groups with respect to body mass index (BMI), baseline IGRA results, and self-reported history of type II diabetes. Those who developed TB were found to have more frequently reported a prior history of TB compared to those who stayed healthy (*P* = 0.004).

**TABLE 1:** Baseline characteristics of 40 individuals selected for Agilent Human microRNA Microarray testing^*a*^

Variable	TB group	LTBI control group	*P*
Total^*b*^, *n*	20	20	
			
Gender, *n* (%)			
Female	10 (50.00)	10 (50.00)	1.000^*c*^
Male	10 (50.00)	10 (50.00)	
			
Age (yrs), *n* (%)			
<40	1 (5.00)	1 (5.00)	1.000^*d*^
40–59	5 (25.00)	6 (30.00)	
≥60	14 (70.00)	13 (65.00)	
			
BMI (kg/m^2^), *n* (%)			
<18.5	3 (15.00)	1 (5.00)	0.633^*d*^
18.5–24	12 (60.00)	15 (75.00)	
≥24	5 (25.00)	4 (20.00)	
			
Self-reported history of type II diabetes, *n* (%)			
No	19 (95.00)	19 (95.00)	1.000^*d*^
Yes	1 (5.00)	1 (5.00)	
			
Self-reported history of household close contacts, *n* (%)			
No	18 (90.00)	18 (90.00)	1.000^*d*^
Yes	2 (10.00)	2 (10.00)	
			
History of prior TB, *n* (%)			
Without	7 (35.00)	16 (80.00)	0.004^*c*^
With	13 (65.00)	4 (20.00)	
			
Baseline TST (mm), median (Q25–Q75)	13.50 (8.75–24.50)	18.75 (14–24.75)	0.144^*e*^
Baseline IFN-γ (IU/mL), median (Q25–Q75)	1.38 (0.53–4.83)	3.06 (1.09–7.65)	0.122^*e*^

a BMI, body mass index; TB, tuberculosis; Q25, 25% quantile; Q75, 75% quantile; TST, tuberculin skin test; IFN-γ, interferon gamma.

b Data might not sum to total due to missing data.

c Obtained by χ^2^ test.

d Obtained by Fisher’s exact test.

e Obtained by Wilcoxon rank-sum test.

### MiRNA microarray analysis of serum miRNAs.

**FIG 1:**
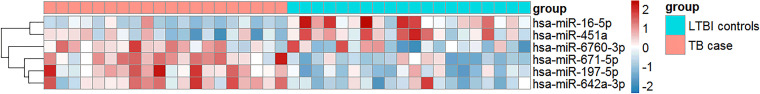
Hierarchical clustering of circulating microRNAs (miR) between tuberculosis (TB) patients and latent tuberculosis infection (LTBI) controls. Red indicates high relative expression and blue indicates low relative expression. Horizontal axis represents each sample: the left 20 are TB patients and the right 20 are LTBI subjects. TB cases are indicated by red rectangles, LTBI controls are indicated by blue rectangles. Expression levels of *hsa-miR-197-5p*, *hsa-miR-671-5p*, *hsa-miR-6760-3p*, and *hsa-miR-642a-3p* were higher, while those of *hsa-miR-16-5p* and *hsa-miR-451a* were lower.

As shown in Fig. 1, the baseline circulating levels of *hsa-miR-197-5p* (*P* < 0.001), *hsa-miR-671-5p* (*P* < 0.001), *hsa-miR-6760-3p* (*P* < 0.001), and *hsa-miR-642a-3p* (*P* < 0.001) were higher, while those of *hsa-miR-16-5p* (*P* < 0.001) and *hsa-miR-451a* (*P* = 0.009) were lower in the case group than in the controls.

### Real-time quantitative PCR analysis of candidate miRNAs.

Demographic information between the two groups for further real-time quantitative PCR (RT-qPCR) verification is given in Table S1 in the supplemental material. Individuals with previous TB history (*P* < 0.001) and lower BMI (*P* = 0.017) were more likely to develop TB. Because levels of *hsa-miR-642a-3p* were not successfully detected by RT-qPCR, they were not included for further data analysis. As shown in Table 2, there were no significant differences in the expression levels of *hsa-miR-197-5p* and *hsa-miR-6760-3p*. The expression level of *hsa-miR-671-5p* was inconsistent with the microarray data. In line with the microarray findings, the baseline circulating levels of *hsa-miR-16-5p* and *hsa-miR-451a* were confirmed to be significantly lower (*P* < 0.001) in the case group than in the control group.

**TABLE 2:** Selected microRNAs identified by microarray for RT-qPCR verification^*a*^

miRNA	miRNA expression levels for TB cases vs LTBI controls					
	Agilent microarray results (case group, *n* = 20; control group, *n* = 20)			RT-qPCR results (case group, *n* = 73; control group, *n* = 77)		
	Fold-change	*P* ^*b*^	Regulation	Fold-change	*P* ^*b*^	Regulation
*hsa-miR-16-5p*	0.27	<0.001	Down	0.45	<0.001	Down
*hsa-miR-197-5p*	2.59	<0.001	Up	0.99	0.482	NA
*hsa-miR-451a*	0.42	0.009	Down	0.22	<0.001	Down
*hsa-miR-671-5p*	2.81	<0.001	Up	0.63	0.005	Down
*hsa-miR-6760-3p*	2.77	<0.001	Up	2.08	0.043	Up

a RT-qPCR, real-time quantitative PCR; LTBI, latent tuberculosis infection; miRNA, microRNA; TB, tuberculosis; NA, not applicable.

b For Wilcoxon rank-sum test, *P* < 0.01 was considered statistically significant.

### Performance of two selected miRNAs on identifying active TB development.

**FIG 2:**
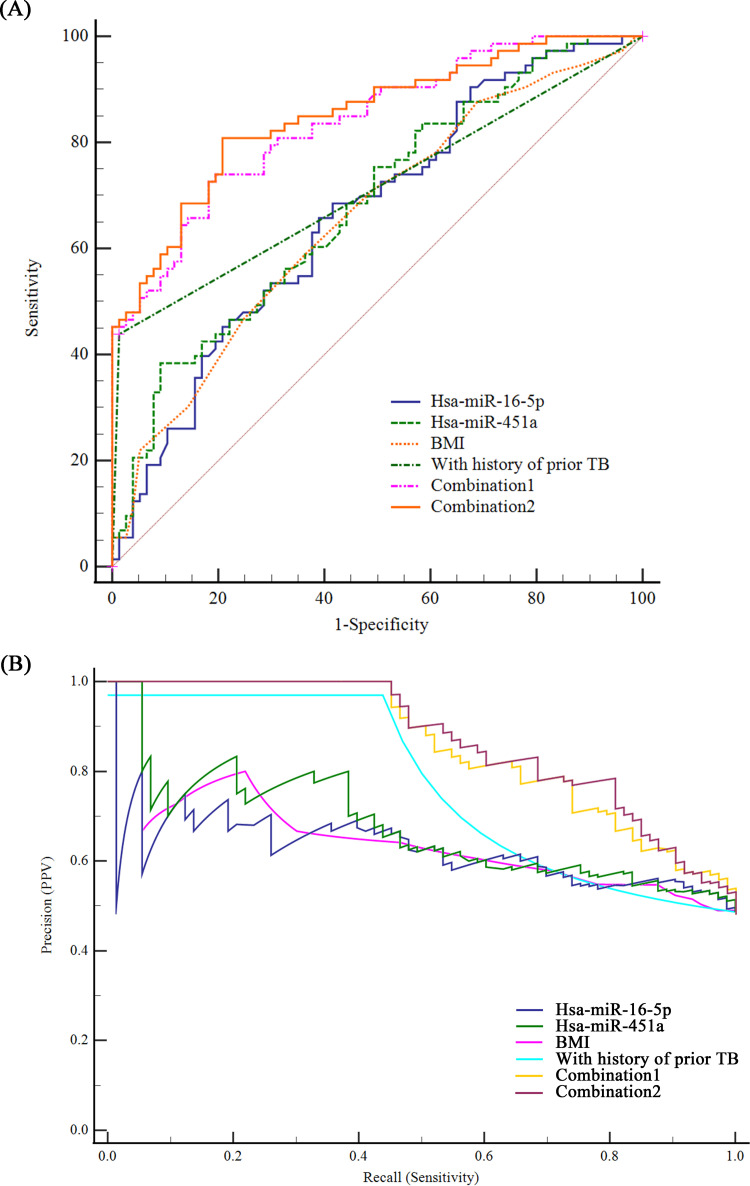
Performance of microRNA(miR) for predicting tuberculosis development. Area under the receiver operator characteristic curves (AUCs, panel A) and precision-recall curves (PRCs, panel B) of body mass index (BMI), with history of prior TB, *hsa-miR-16-5p*, *hsa-miR-451a*, combination 1 (*hsa-miR-16-5p* + BMI + with history of prior TB), and combination 2 (*hsa-miR-451a* + BMI + with history of prior TB) were performed to evaluate their performance in predicting TB development.

Receiver operator characteristic (ROC) curve and precision-recall curve (PRC) analysis were conducted to evaluate the performance of selected miRNAs and co-variables for active TB development. The areas under curves (AUCs) of BMI, with history of prior TB, *hsa-miR-16-5p*, and *hsa-miR-451a* for predicting active TB development from LTBI were 0.66 (95% confidence interval [95% CI]: 0.58 to 0.73), 0.71 (95% CI: 0.63 to 0.78), 0.66 (0.58 to 0.74) and 0.68 (0.60 to 0.76); and the PRCs of these were 0.65 (0.55 to 0.74), 0.77 (0.71 to 0.83), 0.63 (0.52 to 0.71), and 0.67 (0.57 to 0.76), respectively. After consideration of the co-variables (with history of prior TB and BMI), the combined AUCs and PRCs increased to 0.84 (95% CI:0.77 to 0.89), 0.86 (0.79 to 0.90) and 0.85 (0.79 to 0.90), 0.87 (0.81 to 0.91) for *hsa-miR-16-5p* and *hsa-miR-451a*, respectively (Fig. 2). The adjusted AUCs for *hsa-miR-16-5p* and *hsa-miR-451a* were 0.68 and 0.78 after controlling for the influence of two co-variables (Fig. S2 in the supplemental material). Predictive performances of the miRNAs, alone and in different combinations, are shown in Table 3; *hsa-miR-451a* alone showed the highest specificity of 90.91% (95% CI: 82.40 to 95.53%) but the lowest sensitivity of 38.36% (95% CI: 28.05% to 49.83%). *hsa-miR-451a* combined with BMI and with prior history of TB presented the best performance, with a sensitivity of 80.82% (95% CI: 70.34 to 88.22%) and an acceptable specificity of 79.22% (68.88 to 86.78%), with a Youden index of 0.600.

**TABLE 3:** Predicting value of microRNAs on active TB development^*a*^

MicroRNAs	Youden index	Sensitivity, % (95% CI)	Specificity, % (95% CI)
*hsa-miR-16-5p*	0.269	68.49 (57.14 to 78.00)	58.44 (47.29 to 68.79)
*hsa-miR-451a*	0.293	38.36 (28.05 to 49.83)	90.91 (82.40 to 95.53)
*hsa-miR-16-5p* + *hsa-miR-451a*	0.293	38.36 (28.05 to 49.83)	90.91 (82.40–95.53)
*hsa-miR-16-5p* + co-variables^*b*^	0.545	73.97 (62.89–82.66)	80.52 (70.31–87.82)
*hsa-miR-451a* + co-variables^*b*^	0.600	80.82 (70.34–88.22)	79.22 (68.88–86.78)
*hsa-miR-451a* + *hsa-miR-16-5p* + co-variables^*b*^	0.598	76.71 (65.83–84.92)	83.12 (73.23–89.86)

a TB, tuberculosis; CI, confidence interval.

b Youden index and corresponding sensitivity and specificity were calculated based on predicted probability, generated by putting co-variables (BMI, with prior history of TB) and *hsa-miR-16-5p* or *hsa-miR-451a* together in a logistic regression model.

### Target gene prediction and miRNA-Gene network construction.

**FIG 3:**
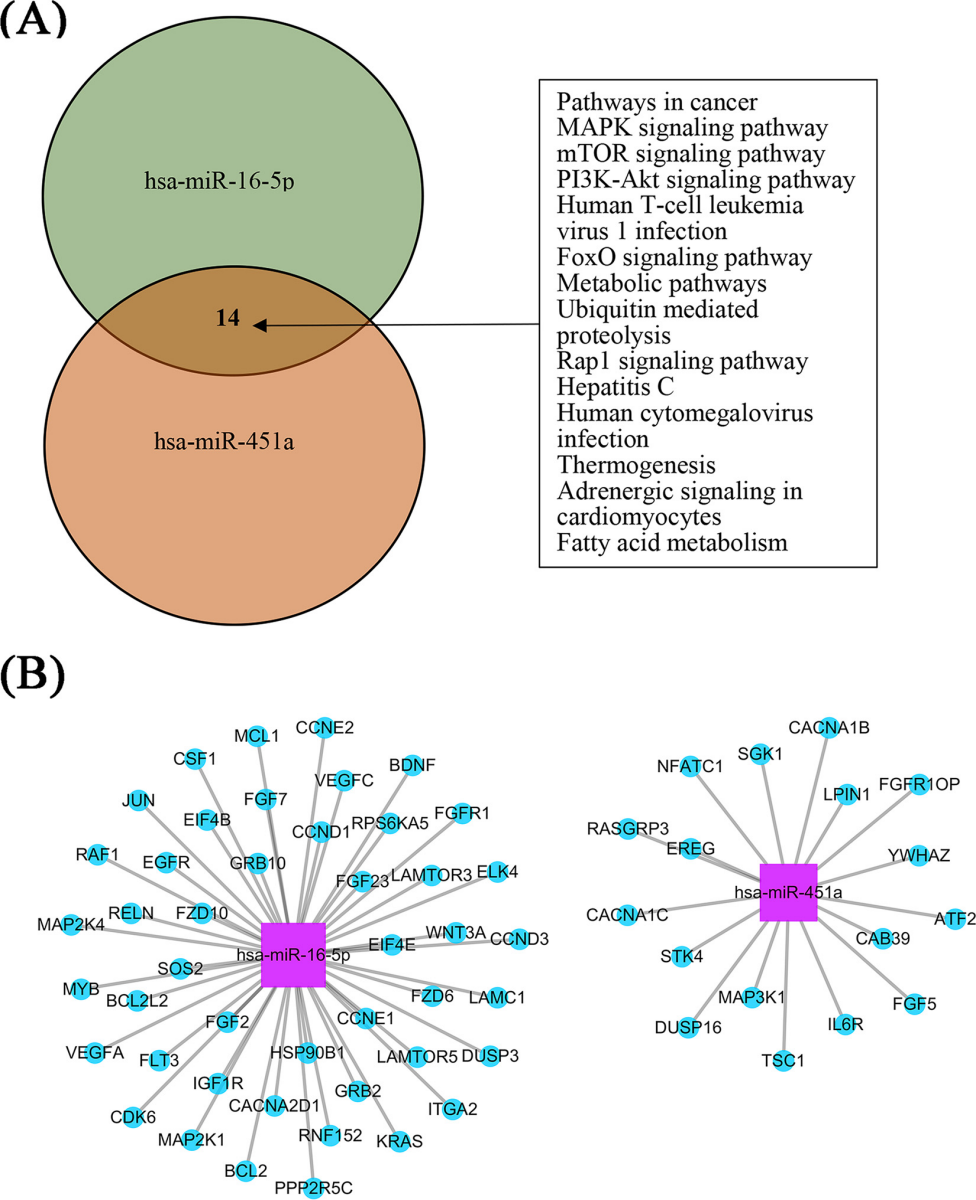
Bioinformatics analysis for *hsa-miR-16-5p* and *hsa-miR-451a*. Kyoto Encyclopedia of Genes and Genomes (KEGG) enrichment analysis was used to annotate and classify the functions of target genes of the selected miRNAs in the pathways. (A) A total of 14 overlapping signaling pathways for the two miRNAs were obtained by VENNY analysis. (B) Target genes involved in the mitogen-activated protein kinase (MAPK), mammalian (mechanistic) target of rapamycin (mTOR), and phosphatidylinositol 3′-kinase–Akt (PI3K-Akt) signaling pathways were selected for miRNA-gene network construction. Pink color represents miRNAs and blue color represents genes.

As depicted in Fig. 3A, Kyoto Encyclopedia of Genes and Genomes (KEGG) enrichment analysis revealed that the target genes of *hsa-miR-16-5p* and *hsa-miR-451a* were potentially involved in 14 common signaling pathways. In addition, 45 and 17 target genes of *hsa-miR-16-5p* and *hsa-miR-451a*, possibly related to TB development, from the mitogen-activated protein kinase (MAPK), mammalian (mechanistic) target of rapamycin (mTOR), and phosphatidylinositol 3′-kinase–Akt (PI3K-Akt) signaling pathways were selected for miRNA-gene network construction. Common target genes from the MAPK pathways for these two miRNAs were found (Fig. 3B).

## DISCUSSIONS

As we knew previously, the performance of circulating miRNAs in predicting the development of active TB has been rarely studied by means of prospective study designs. This study was based on a prospective study designed to track the development of active TB in LTBI, and the baseline levels of circulating miRNAs between those who developed TB and those remained free of TB throughout the study were retrospectively detected and compared. We found that the baseline levels of *hsa-miR-16-5p* and *hsa-miR-451a* were significantly lower in subjects who developed active TB compared to those who stayed healthy. *hsa-miR-451a* showed considerable potential for predicting active TB development from LTBI.

Exploring biomarkers that could help identify subjects with a high likelihood of progressing to active TB from LTBI are meaningful for practicing precise intervention. A study conducted by Miotto et. al. (10) reported that the expression levels of miR-451 and miR-16 were significantly upregulated in TB patients compared with that in healthy controls. A subsequent cohort study in India evaluated the levels of selected miRNAs in serum from TB patients and their levels during therapy, and found the miR-16 level was significantly elevated in TB patients compared to that in uninfected controls, returning to the levels observed in healthy subjects after treatment (11). In addition to studies of serum miRNAs, studies conducted in host peripheral blood mononuclear cells or macrophages also found the expressions of miR-451 and miR-16 to be significantly upregulated in active TB patients compared with that in LTBI patients and in healthy controls (12, 13). In our study, individuals with LTBI with lower levels of *hsa-miR-16-5p* and *hsa-miR-451a* were found to be increased risk of developing active disease. The nested case-control study design could provide stronger evidence for causal inference than traditional case-control or cross-sectional design (7). Several studies have explored gene expression screening methods to identify the signatures of active TB disease risk by means of prospective study design as well (14–16).

Our results needed to be validated by further observational studies in various populations as well as by function and action mechanism research. The two miRNAs identified in our study may be involved in modulating the balance between host immune protection and *M.tb* persistent infection. It has been reported that miR-16 is involved in regulating the innate immune system by targeting IκB kinase α (IKK α) mRNA, which plays important roles in the noncanonical nuclear factor kappa B (NF-κB) signaling pathway. This pathway prevents the new macrophage from becoming over-activated, which might increase the susceptibility to TB by repressing the activation of NF-κB target genes (17). *hsa-miR-451* is well known as a promising candidate miRNA involved in responses to various pathogen infections, including influenza infections (18, 19). According to KEGG pathway prediction, 14 common pathways are shared by these two candidate miRNAs. Among them, the MAPK, mTOR, and PI3K-Akt signaling pathways have been found to be involved in the host response to TB disease (20–23). Matrix metalloproteinases (MMPs), downstream effector molecules of the MAPK signaling pathway, induce lung tissue remodeling and contribute to early TB granuloma formation (24, 25). Additionally, a variety of MMP inhibitors have been used to study the immunomodulatory effects of MMPs in *M.tb* infection, and have shown the immunomodulatory roles of MMPs in *M.tb* pathogenesis (26, 27). PI3K/Akt/mTOR signaling was reported to be involved in the epithelial cell-modulated, *M.tb*-activated Toll-like receptor signaling pathway, and observational study provided evidence of an association between mTOR polymorphism and TB susceptibility (22, 23). Although no common target gene was found for the two miRNAs, MAP2k4 and MAP2k1 from *hsa-miR-16-5p* and MAP3k1 from *hsa-miR-451a* were found, further indicating the importance of the MAPK pathway in TB development. Further mechanism studies are needed to validate this speculation.

When interpreting our results, several limitations should be kept in mind. First, to explore the most promising potential miRNAs related to TB development, strict criteria were used to select candidate miRNAs for further RT-qPCR verification. Thus, some relevant miRNAs might have been over-excluded. Second, because the level of *hsa-miR-642a-3p* was not successfully detected by RT-qPCR, primer design or sample quality might have influenced our results. Third, for *hsa-miR-451a* and *hsa-miR-16-5p*, the false discovery rates were higher than 0.05 after adjustment for multiple comparisons in microarray analyses. Given the reported evidence on their association with TB disease (13, 14), we still included them in further RT-qPCR verification and observed positive relations with a larger sample size. As we retrospectively calculated, the power of the present study to identify significance was 99% for *hsa-miR-451a* and 96% for *hsa-miR-16-5p*, respectively. Fourth, the distribution of prior TB history and BMI was uneven between the two groups. We performed an adjusted AUC by controlling the two variables, and *hsa-miR-451a* still showed substantial prediction potential. Nevertheless, the influence of other uninvestigated factors on the results cannot be ignored. Fifth, although randomly selected samples were used for RT-qPCR validation, they were not collected from an independent sample set. Therefore, further studies are needed to validate our results in the future for external validation.

### Conclusions.

In conclusion, circulating levels of *hsa-miR-451a* showed good potential for distinguishing individuals at risk for developing active TB from LTBI in a Chinese population. Our findings need to be validated in various populations and the underlying mechanisms need to be further explored.

## MATERIALS AND METHODS

### Study population.

The present study was based on a prospective study aiming to track the development of active TB from LTBI in residents of rural China between 2013 and 2018. Detailed information on the study design has been published elsewhere (9, 28). Briefly, 21,022 rural residents aged 5 years or older and without HIV infections were screened for TB infection testing by both IGRA and TST in a baseline survey in 2013. Subsequently, 7,505 individuals with baseline positive results by IGRA or TST (≥10 mm) were identified and followed for 5 years to track active TB development and divided into two groups, a TB case group and an LTBI control group, according to whether TB was detected (Diagnosis of Pulmonary Tuberculosis [WS 288-2017]). A nested case-control design was used to retrospectively detect and compare baseline levels of circulating miRNAs between the two groups.

A total of 117 TB incidence cases were identified during the 5-year follow-up among 7,505 LTBI patients, as previously described (28). As shown in Fig. S1, 20 TB cases and 20 age- and gender-matched LTBI controls with both positive IGRA and TST results were randomly selected for Agilent Human miRNA detection to estimate the circulating levels of 2,549 miRNAs in each study group. This step aimed to detect thousands of miRNAs simultaneously using a high-throughput Agilent array technique. Next, differently expressed miRNAs identified by microarray were selected for further verification in a larger sample size (73 cases who developed active TB and 77 controls who remained LTBI) using real-time quantitative PCR with low cost. LTBI controls were matched with cases by study site (four study sites), gender (male and female), and age group (<40 years, 40 to 50 years, 50 to 60 years, 60 to 70 years, 70 to 80 years, ≥80 years). Written informed consent was obtained from each participant, and the study was conducted in accordance with the Declaration of Helsinki. The ethics committees of the Institute of Pathogen Biology of the Chinese Academy of Medical Sciences approved this study.

### RNA isolation and purification.

For each participant, 2 mL serum samples were collected at the baseline survey. Total RNA, including miRNAs, was extracted and purified from serum using a Qiagen Serum/Plasma kit (Qiagen no. 217184) following the manufacturer’s instructions and checked (at least 120 μg) by a NanoDrop ND-2000 spectrophotometer (Thermo Fisher Scientific, US). (see Tables S2 and S3 for total RNA).

### miRNA microarray hybridization.

miRNA microarray assays were performed using the Agilent Human miRNA Microarray platform (design ID: 070156) at Shanghai Biotechnology Co., Ltd. (Shanghai, China). The microarray contains probes for 2,549 human miRNAs sequences from the Sanger miRBase (release 21.0, University of Manchester, Manchester, United Kingdom). Labeling and hybridization were performed according to the protocols in the Agilent miRNA microarray system.

### Computational analysis of miRNA microarray data.

Slides were scanned by an Agilent Microarray Scanner and Feature Extraction software version 10.7 with default settings. Raw data were normalized by the Quantile algorithm and converted to a logarithmic value with base 2 (log_2_). Fold-change was determined by comparing log_2_ ratios between the two groups. Significantly expressed miRNA were defined by a fold change of ≥2 or ≤0.5, *P* < 0.05. In least one group, the probe signal should be significantly higher than the background signal for at least 90% samples.

### RT-qPCR analysis.

Statistically differently expressed miRNAs were further verified by TaqMan microRNA assays according to the manufacturer’s protocol. RT-qPCR analysis was performed at Shanghai Biotechnology Co., Ltd. (Shanghai, China) using a QuantStudio 5 Real-Time PCR system (Applied Biosystems, Waltham, MA) under the following conditions: 95°C for 10 min, followed by 40 cycles at 95°C for 15 s and 60°C for 1 min. *cel-miR-39* was used as an endogenous control for normalization in this study. All assays were carried out in triplicate and mean cycle threshold (*C_T_*) was calculated to determine relative gene expression levels. The relative expression levels of each target miRNA (log_2_ relative level) were calculated according to differences in *C_T_* values between the target miRNAs and *cel-miR-39* by using the 2^-ΔΔ^*^CT^* method.

### Bioinformatics analyses of differentially expressed miRNA target genes.

Bioinformatics analyses were used to predict gene targets, followed by Ingenuity Pathway Analysis (Ingenuity Systems, www.ingenuity.com). Kyoto Encyclopedia of Genes and Genomes (KEGG) pathway enrichment analysis was used to annotate and classify functions of the target genes of the differently expressed miRNA in the pathways, and Cytoscape was used to construct the miRNA-gene network.

### Statistical analysis.

The Statistical Analysis System (SAS 9.4 for Windows; SAS Institute Inc., NC) and MedCalc Statistical Software version 20.027 (MedCalc Software Ltd., Ostend, Belgium) were used for analyses. The SBC Analysis System using functions of the R package was used for microarray data analysis. Fold changes in expression signals between the two groups were calculated from the normalized values. Chi-square and Fisher’s exact tests were used to compare the distributions of categorical variables. Variables with a *P* of <0.05 in the univariate analysis were included in unconditional multiple logistic regression analyses, and associations were assessed with an odds ratio (OR) and a 95% confidence interval (95% CI). Wilcoxon rank-sum tests were used to compare miRNA levels. When multiple-comparison testing was needed, the significance level was adjusted using the Bonferroni method, dividing the significance level (0.05) by the number of simultaneous tests. Hierarchical clustering was performed with Pearson’s correlation for differentially expressed miRNAs. Receiver operator characteristic curve analysis, presented with area under the ROC curve and precision analysis presented with precision-recall curve analysis, were performed to evaluate the performance of a combination of miRNAs with co-variables for predicting disease development. Covariate-adjusted ROC analysis and the corresponding adjusted AUCs were also conducted after controlling for potential confounders using R software version 4.0.5 (R Foundation for Statistical Computing, Vienna, Austria) with the analysis packages ROCt version 0.9.5. VENNY analysis was conducted to find the intersection of target signaling pathways for the selected miRNAs. A *P* value of <0.05 was considered statistically significant.

### Data availability.

Data supporting the findings of this study are available within the article and its supplemental material. Raw data can be uploaded upon request.
