# Supplementary material for: The Association between Circulating microRNAs and the Risk of Active Disease Development from Latent Tuberculosis Infection: a Nested Case-Control Study
Source: Microbiol Spectr. 2022 Apr 18;10(3):e02625-21. doi: 10.1128/spectrum.02625-21 (PMC9241859; doi:10.1128/spectrum.02625-21)
Supplement: SUPPLEMENTAL FILE 1 — Supplemental material. Download spectrum.02625-21-s001.pdf, PDF file, 0.5 MB [file spectrum.02625-21-s001.pdf]

Supporting Information

The association between circulating microRNAs and the risk of active disease development from latent tuberculosis infection: a nested case-control study

Henan Xin[1†], Xuefang Cao[1†], Ying Du[1†], Jiaoxia Yan[2†], Rui He[3], Zisen Liu[2], Haoran Zhang[1], Yijun He[1], Bin Zhang[2], Dakuan Wang[2], Ling Guan[3], Fei Shen[3], Boxuan Feng[1], Zhusheng Quan[1], Yongpeng He[1], Jianmin Liu[3], Qi Jin[1], *Shouguo Pan[2], *Lei Gao[1]

1. NHC Key Laboratory of Systems Biology of Pathogens, Institute of Pathogen Biology, and Center for Tuberculosis Research, Chinese Academy of Medical Sciences and Peking Union Medical College, Beijing 100730, P.R. China.
2. Center for Diseases Control and Prevention of Zhongmu, Zhengzhou 451450, China.
3. The Sixth People's Hospital of Zhengzhou, Zhengzhou 400060, China.

[†] Contributed equally

Correspondence: Prof. Lei Gao, NHC Key Laboratory of Systems Biology of Pathogens, Institute of Pathogen Biology, Chinese Academy of Medical Sciences and Peking Union Medical College, No 9 Dong Dan San Tiao, Beijing 100730, China. Email: gaolei@ipbcams.ac.cn. Prof. Shouguo Pan, Center for Diseases Control and Prevention of Zhongmu, No. 1106, Youth West Road, Zhongmou County, Zhengzhou 451450, China., Email: psgzmcdc@163.com

Table S1 Baseline characteristics of 150 individuals selected for RT-qPCR verification

Variables	TB cases	LTBI controls	P value	OR (95%CI)
**Total***, n	73	77		
**Gender**, n (%)				
Female	23 (31.51)	25 (32.47)	0.900#	
Male	50 (68.49)	52 (67.53)		
**Age (years)**, n (%)				
<40	5 (6.85)	5 (6.49)	0.984#	
40-59	19 (26.03)	21 (27.27)		
≥60	49 (67.12)	51 (66.23)		
**BMI (kg/m^2)** , n (%)				
<18.5	8 (10.96)	3 (3.90)	**0.017#**	
18.5-24	46 (63.01)	38 (49.35)		
≥24	19 (26.03)	36 (46.75)		
Mean±SD	22.1±3.24	24.0±3.54	0.001&	**0.88 (0.78-0.99)**
**Self-reported history of type 2 diabetes**, n (%)				
No	66 (90.41)	70 (90.91)	0.917#	
Yes	7 (9.59)	7 (9.09)		
**Self-reported history of household close contacts**, n (%)				
No	64 (87.67)	75 (97.40)	**0.022** #	Ref
Yes	9 (12.33)	2 (2.60)		3.68 (0.64-21.15)
**History of prior TB†**, n (%)				
Without prior TB	41 (56.16)	76 (98.70)	**<0.001** #	Ref
With prior TB	32 (43.84)	1 (1.30)		**50.90 (6.63-390.65)**
**Baseline TST (mm)**				
Median, Q25-Q75	23.00 (18.00-29.50)	20.50 (15.50-26.00)	0.082†	
**Baseline IFN-γ（IU/ml）**				
Median, Q25-Q75	2.46 (1.35-5.34)	2.26 (1.21-5.13)	0.700†	

Abbreviation: CI=confidence interval; BMI=Body mass index; TB=tuberculosis; Q25=25% quantile; Q75=75% quantile; TST= tuberculin skin test; OR=odds ratio; RT-qPCR, real-time Quantitative PCR. *Data might not sum to total because of missing data. †p for Wilcoxon rank sum test. # p for χ^2 test. &p for T test.

Table S2 Total RNA for 40 samples used for microarray

Reagent Name	Qiagen serum/Plasma Kit-Qiagen#217184	Reagent Lot#	154035945	Number of samples: 40
**No.**	**Sample Name**	**Con. (ng/μL)**	**Vol. (μL)**	**Total (μg)**
1	21217301a	5.6	30	0.168
2	22205801a	13	30	0.39
3	52028201a	8	30	0.24
4	51187401a	7.1	30	0.213
5	51187403a	10.7	30	0.321
6	51162201a	6	30	0.18
7	51146401a	5.6	30	0.168
8	51140201a	4.7	30	0.141
9	51081606a	5.6	30	0.168
10	51081801a	6.3	30	0.189
11	51102306a	5.7	30	0.171
12	52080102a	5.1	30	0.153
13	51226901a	5.6	30	0.168
14	51261102a	6.9	30	0.207
15	52047301a	7.4	30	0.222
16	52009601a	4.4	30	0.132
17	52067001a	5.9	30	0.177
18	51202001a	7.2	30	0.216
19	51243702a	5.2	30	0.156
20	31095601a	7.4	30	0.222
21	40104002	12.6	30	0.378
22	21109301	10	30	0.3
23	21411601	12	30	0.36
24	21215201	11.9	30	0.357
25	40102943	12.2	30	0.366
26	40106592	10.1	30	0.303
27	21305303	8	30	0.24
28	21100502	8.9	30	0.267
29	40106030	5.3	30	0.159
30	21428301	6.3	30	0.189
31	21110601	10.3	30	0.309
32	40201081	5.8	30	0.174
33	21216801	7.1	30	0.213
34	21111901	6.3	30	0.189
35	40201061	7.4	30	0.222
36	22314902	6.7	30	0.201
37	40103040	15.8	30	0.474
38	21414001	11.2	30	0.336
39	22511304	5	30	0.15
40	22102601	5.4	30	0.162

Table S3 Total RNA for 150 samples used for RT-qPCR

Reagent Name	Qiagen serum/Plasma Kit-Qiagen#217184	Reagent Lot#	160044181	Number of samples: 150
**No.**	**Sample Name**	**Con. (ng/µL)**	**Vol. (µL)**	**Total (µg)**
1	14002201	5.8	30	0.174
2	14013901	6	30	0.18
3	16004601	6.6	30	0.198
4	16024201	7.2	30	0.216
5	12000301	7.6	30	0.228
6	12038601	5.3	30	0.159
7	12023101	4.7	30	0.141
8	12004401	5.9	30	0.177
9	12025801	6.8	30	0.204
10	12020303	8.9	30	0.267
11	12005101	6.9	30	0.207
12	13027302	6.8	30	0.204
13	13004101	5	30	0.15
14	13016304	6.1	30	0.183
15	13017902	6.4	30	0.192
16	13011604	5.1	30	0.153
17	13001902	7	30	0.21
18	13014501	10.4	30	0.312
19	13022807	12.2	30	0.366
20	11023301	8.6	30	0.258
21	11014104	6.5	30	0.195
22	11027201	6.6	30	0.198
23	11041103	6.4	30	0.192
24	16026901	12.3	30	0.369
25	16040904	9.4	30	0.282
26	14009201	7.6	30	0.228
27	16006801	6.7	30	0.201
28	15029201	7.3	30	0.219
29	15013403	8.3	30	0.249
30	15043101	5.4	30	0.162
31	15024502	5.8	30	0.174
32	21417102	6.4	30	0.192
33	21206505	8.7	30	0.261
34	23304001	5.3	30	0.159
35	23317002	6.6	30	0.198
36	22512301	6.1	30	0.183
37	21217301	6	30	0.18
38	21205105	7.2	30	0.216

39	22205801	6.1	30	0.183
40	32008403	5.6	30	0.168
41	32056403	5.4	30	0.162
42	31022101	7.9	30	0.237
43	31035803	5.5	30	0.165
44	31989503	6.1	30	0.183
45	31139101	5.6	30	0.168
46	32011501	5.5	30	0.165
47	32070401	10	30	0.3
48	31045004	7.8	30	0.234
49	32069705	6.7	30	0.201
50	32016903	9.3	30	0.279
51	31095601	10.6	30	0.318
52	51081606	7.6	30	0.228
53	52047301	9.3	30	0.279
54	51063202	5.4	30	0.162
55	51140201	16.5	30	0.495
56	52080102	8.2	30	0.246
57	51083202	8.1	30	0.243
58	51261102	7.1	30	0.213
59	51043605	7.9	30	0.237
60	51243702	5.9	30	0.177
61	51260603	7.7	30	0.231
62	52067001	8.2	30	0.246
63	53001603	7	30	0.21
64	51000201	11.3	30	0.339
65	51162201	9.3	30	0.279
66	51187403	6.1	30	0.183
67	51281101	8.6	30	0.258
68	51044801	6.6	30	0.198
69	51203901	8.4	30	0.252
70	51244401	6.5	30	0.195
71	51226901	9.5	30	0.285
72	51286501	7	30	0.21
73	51061601	6.2	30	0.186
74	11022301	5.8	30	0.174
75	11021702	7.8	30	0.234
76	11031602	11.7	30	0.351
77	11032801	6.6	30	0.198
78	11035803	5.9	30	0.177
79	11040903	6.1	30	0.183
80	11002601	8.9	30	0.267
81	11041201	6.4	30	0.192
82	11041403	5.3	30	0.159
83	11041705	6	30	0.18

84	16037703	7.1	30	0.213
85	16038602	5.6	30	0.168
86	11024001	4.6	30	0.138
87	15041901	4.9	30	0.147
88	15039302	10.1	30	0.30
89	15027101	6	30	0.18
90	15035502	6.9	30	0.207
91	15009001	6.4	30	0.192
92	15035102	4.2	30	0.126
93	15007301	6.5	30	0.195
94	15035305	4.5	30	0.135
95	15018001	8.9	30	0.27
96	15037703	5.3	30	0.159
97	15006101	7	30	0.21
98	15019701	7.9	30	0.24
99	15030102	7	30	0.22
100	13027802	9.4	30	0.28
101	11032201	4.5	30	0.135
102	14016301	7.4	30	0.22
103	14004204	4.1	30	0.123
104	14033801	5.7	30	0.171
105	16026301	5.5	30	0.165
106	12021502	6.8	30	0.204
107	13004103	5.4	30	0.162
108	22320602	6.1	30	0.183
109	22326108	4.8	30	0.144
110	23335104	5.1	30	0.153
111	21200302	6.3	30	0.189
112	21403001	5	30	0.15
113	21410903	9.1	30	0.28
114	22208102	4.8	30	0.144
115	22407201	9.3	30	0.28
116	22105804	4.1	30	0.123
117	22308202	6.1	30	0.183
118	31090704	5.3	30	0.159
119	31084901	6.8	30	0.20
120	31113101	6.7	30	0.20
121	31136503	10.5	30	0.32
122	32065001	14.1	30	0.423
123	32070001	6.1	30	0.183
124	32054403	5.6	30	0.168
125	31020501	8.3	30	0.25
126	31042001	8.7	30	0.27
127	52044201	7.8	30	0.23
128	52003901	4.7	30	0.141

129	52065505	4.2	30	0.126
130	52027601	4.2	30	0.126
131	52028605	4.9	30	0.147
132	51205401	5	30	0.15
133	51208203	5.4	30	0.162
134	51102902	4.5	30	0.135
135	51084306	5.7	30	0.171
136	51220306	8.4	30	0.25
137	51162401	7.8	30	0.24
138	51142902	5.4	30	0.162
139	51081603	7.5	30	0.225
140	51062101	6.6	30	0.20
141	51121601	7.9	30	0.23
142	51124203	4.6	30	0.138
143	51081701	6.5	30	0.195
144	53002801	6.8	30	0.21
145	51261702	8.7	30	0.26
146	51283002	5.3	30	0.159
147	51200202	6.6	30	0.20
148	51245202	6.4	30	0.192
149	51244105	4.8	30	0.144
150	51243501	5.3	30	0.159

RT-qPCR, real-time Quantitative PCR

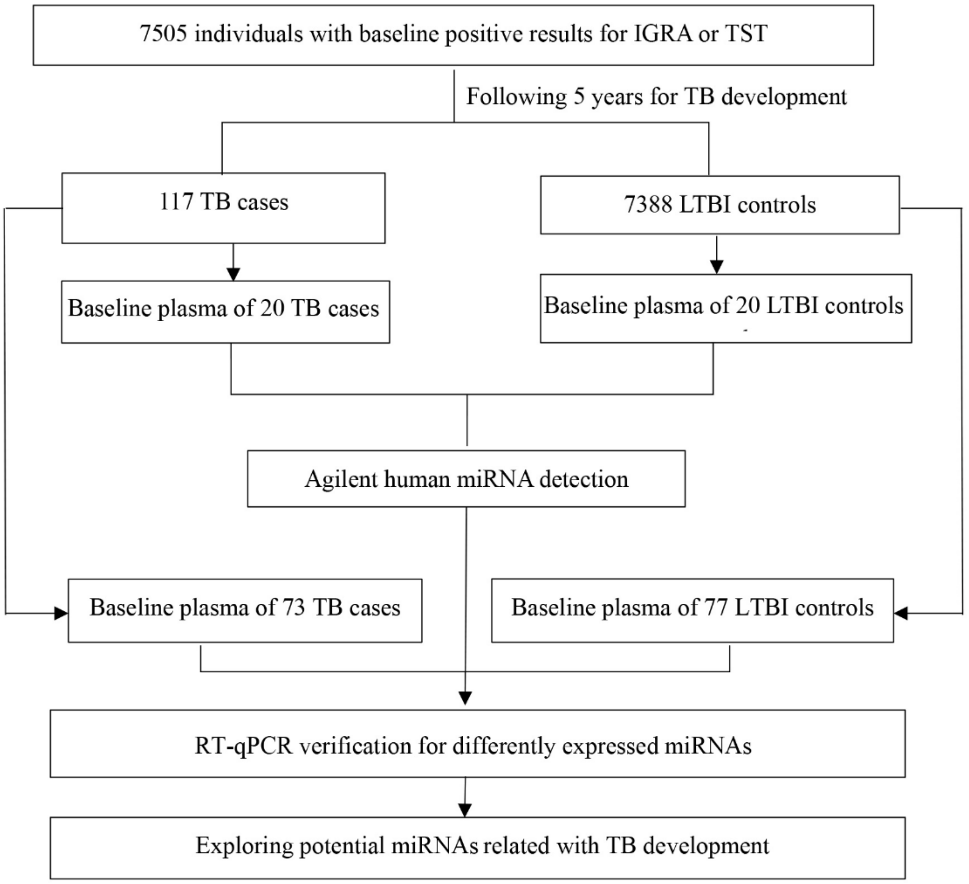

Supplementary Figure 1 Flow chart

A total of 7505 individuals with baseline positive results for IGRA or TST (≥10mm) were followed up 5 years for active TB development. During the five years, 117 TB cases were identified. We first selected 20 TB cases from the 117 cases and 20 age and sex-matched LTBI controls with both positive IGRA and TST results for Agilent Human miRNA Microarray platform interrogating 2549 miRNAs. Six differently expressed miRNA (hsa-miR-16-5p, hsa-miR-197-5p, hsa-miR-451a, hsa-miR-630, hsa-miR-671-5p, hsa-miR-6760-3p) were selected for further RT-qPCR verification among 150 samples (73 TB cases with IGRA+/TST+ results and age-and sex-matched 77 LTBI controls with IGRA+/TST+ results). Abbreviation: IGRA=interferon-gamma release assays; LTBI=latent tuberculosis infection; miRNA=microRNA; RT-qPCR=Real-time quantitative PCR; TB=tuberculosis; TST=tuberculin skin test.

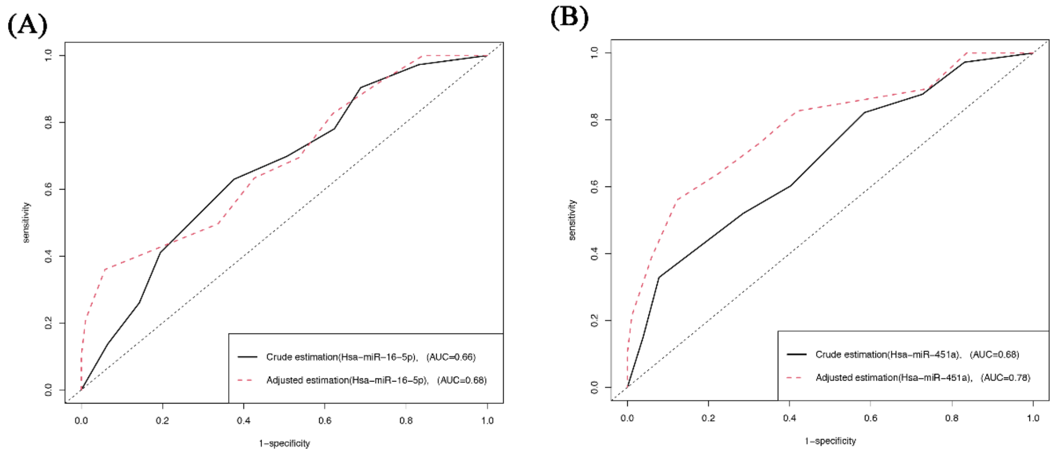

Supplementary Figure 2 Adjusted AUCs for hsa-miR-16-5p and hsa-miR-451a

Adjusted AUC for hsa-miR-16-5p (A) and hsa-miR-451a (B) were showed after adjusting the influence of two variables, BMI and with history of prior TB. Hsa-miR-451a showed considerable potentiality in predicting TB development from LTBI. Abbreviation: AUC= area under the receiver operator characteristic curve; BMI= body mass indicator; LTBI=latent tuberculosis infection; TB=tuberculosis.
